# Enhancing PAPR and Throughput for DFT-s-OFDM System Using FTN and IOTA Filtering

**DOI:** 10.3390/s22134907

**Published:** 2022-06-29

**Authors:** Xinran Zhuo, Jianxiong Pan, Huwei Wang, Xiangming Li, Neng Ye

**Affiliations:** 1 School of Cyberspace Science and Technology, Beijing Institute of Technology, Beijing 100081, China; xrzhuo@bit.edu.cn; 2 Science and Technology on Communication Networks Laboratory, Shijiazhuang 050081, China; 3 School of Information and Electronics, Beijing Institute of Technology, Beijing 100081, China; panjianxiong@bit.edu.cn (J.P.); whw@bit.edu.cn (H.W.)

**Keywords:** waveform, DFT-s-OFDM, FTN, IOTA filter, PAPR, high frequency

## Abstract

High frequency wireless communication aims to provide ultra high-speed transmissions for various application scenarios. The waveform design for high frequency communication is challenging due to the requirements for high spectrum efficiency, as well as good hardware compatibility. With high flexibility and low peak-to-average power ratio (PAPR), discrete Fourier transformation spreading-based orthogonal frequency division multiplexing (DFT-s-OFDM) can be a promising candidate waveform. To further enhance the spectral efficiency, we integrate faster-than-Nyquist (FTN) signaling in DFT-s-OFDM, and find that the PAPR performance can also be improved. While FTN can introduce increased inter-symbol interference (ISI), in this paper, we deploy an isotropic orthogonal transform algorithm (IOTA) filter for FTN-enhanced DFT-s-OFDM, where the compact time-frequency structure of the IOTA filter can significantly reduce the ISI. Simulation results show that the proposed waveform is capable of achieving good performance in PAPR, bit error rate (BER) and throughput, simultaneously, with 3.5 dB gain in PAPR and 50% gain in throughput.

## 1. Introduction

High frequency, i.e., millimeter wave (mmWave) and terahertz (THz) wireless communication technology, which exploits the treasure of large bandwidth in higher frequency, has become a research hotspot with the evolution of B5G and 6G [1,2,3,4]. The aim of high frequency technology is to provide extremely high-speed data rate for various applications, such as vehicular networks, satellite backhauls, extended reality (XR) applications, etc. [5,6,7,8,9,10]. The characteristics of the devices and the propagation environment are significantly different at high frequency. For example, the linear range of the amplifier at high frequency is small, which causes unexpected distortion on the signal waveforms with a large variance [11]. The design of the waveform thus becomes a major concern in enabling high frequency communication.

The designs of waveforms based on orthogonal frequency division multiplexing (OFDM) have been commercialized in 4G/5G, and are now the baseline of 6G high frequency communication [12,13]. Parallel transmission of high speed data is realized in OFDM through time and frequency division multiplexing, which has good resistance to multi-path fading and can support efficient multi-user access. Discrete Fourier transform (DFT) spreading technique has been incorporated in OFDM, termed DFT-s-OFDM, to achieve lower peak-to-average power ratio (PAPR) so as to make it compatible with the low-ability power amplifier, e.g., cellular uplink and ground-to-satellite link. While maintaining the characteristics of single-carrier waveforms, DFT-s-OFDM offers more flexibility compared to the conventional single-carrier system. DFT-s-OFDM has been applied in high frequency communication scenarios [14], which realize multi-user multiplexing when ensuring good PAPR performance. It has also been proved that DFT-s-OFDM can achieve a balance between user scheduling flexibility and computational complexity of channel equalization [15], which enables usage in wireless hotspots with densely distributed user equipment. Recently, waveform variants based on DFT-s-OFDM, e.g., generalized DFT-s-OFDM which redesigns the cyclic prefix (CP) [16,17], have triggered more research interest in achieving lower latency and out-of-band emission. Despite the above variances, achieving the improvement of both the PAPR and throughput at the same time is a challenge. To enhance the spectral efficiency, utilizing high-order modulation is a straightforward method. However, the different amplitudes of modulated symbols leads to larger PAPR, which makes reducing PAPR and enhancing throughput contradictory. As for the methods which achieve lower PAPR, e.g., applying DFT spreading on OFDM, they cannot improve the spectral efficiency. PAPR can be further lowered by methods such as peak clipping. However, this can cause signal distortion and the spectral efficiency is sacrificed. FTN signaling is an efficient method for enhancing the spectral efficiency, with the disadvantage of low transmission reliability due to the additional ISI brought by acceleration.

In order to further enhance the spectral efficiency of transmission, faster-than-Nyquist (FTN) technology has been put forward and introduced into some existing waveform frameworks. Compared to the systems based on Nyquist criteria, FTN signaling can achieve 25% improvement in symbol rate without sacrificing the error rate performance. Basic ideas of incorporating FTN signaling in OFDM-based waveforms are introduced in [18]. FTN also exhibits high compatibility with different modulations, where [19] validates the capacity and SNR gain of FTN-based low-order modulations, and [20] proves the power gains of FTN-based high-order modulations in a multi-carrier system. As for the receiving technology of FTN, Ref. [21] investigates the maximum posterior-based detection of FTN signaling, which achieves up to 186% gains in spectral efficiency compared with its counterpart of Nyquist signaling. To reduce the computational complexity of the FTN receiver, Ref. [22] proposes a deep learning-aided FTN signal detection method for an uplink multi-user transmission scenario. Despite its throughput gain, the major drawback of FTN is that the accelerated symbols will introduce additional inter-symbol interference (ISI), which degrades the transmission reliability [23]. The proposed method provides a candidate solution for jointly enhancing the performance of PAPR and throughput. We first increase the spectral efficiency by integrating FTN with the low-PAPR DFT-s-OFDM. At the same time, we design an IOTA shaping filter to reduce the ISI. The proposed method is simple but efficient, which can improve the performance of PAPR and throughput simultaneously.

In this paper, to enhance the performances of PAPR and throughput simultaneously, we consider the incorporation of FTN with DFT-s-OFDM and aim to tackle the problem of additional ISI caused by FTN. Specifically, we propose an FTN-DFT-s-OFDM waveform based on an isotropic orthogonal transform algorithm (IOTA) filter, which can lower the ISI while keeping the spectral efficiency enhanced. Good focusing ability on both time and frequency domain can be found in IOTA filters, which is vital for reducing the ISI and ICI [24]. Besides, experimental results have shown that IOTA filters help in obtaining higher computational efficiency [25]. By combining the above-mentioned ingredients, the detailed design of the proposed transmission waveform is presented in the following sections. The low ISI characteristic also makes the low-complexity frequency-domain equalization-based method available for reliable detection. Monte Carlo simulation is performed to validate that the joint benefits of deploying IOTA and FTN technologies in DFT-s-OFDM can be obtained with respect to PAPR, bit error rate (BER) and throughput. Different modulation coding schemes (MCSs) have been simulated, where up to 3.5 dB PAPR gain, 50% throughput gain in the high SNR region, can be achieved by the proposed scheme, compared to conventional waveforms.

The rest of this paper is organized as follows. Section 2 of this paper illustrates the signal model and performance metrics used in the simulation process. Next, the proposed IOTA-based FTN-DFT-s-OFDM Waveform Design is introduced in Section 3, including the transmission waveform design and receiver design. Then in Section 4, extensive simulation results of the proposed waveform are shown, with respect to PAPR, BER, and throughput under various MCSs. Finally, Section 5 concludes this paper.

## 2. Signal Model and Performance Metrics

In this section, we consider a general waveform framework, as well as the metrics which can evaluate the performance of a given waveform.

### 2.1. General Waveform Framework

Multi-carrier waveform supports parallel transmission, where high-rate data stream is decomposed into multiple parallel low-rate sub-data streams. Each sub-data stream is independently modulated and superimposed to form the transmission signal.

Orthogonal Frequency Division Multiplexing (OFDM) waveform is a special kind of multi-carrier transmission technology. In the OFDM system, the transceiver filters form the special modulation filters, where the transmitting filter can be realized by IDFT and the receiving filter can be realized by DFT. With the usage of cyclic prefix, OFDM technology solves the problem of multi-path fading with low complexity. The following part describes the general framework of OFDM.

**Figure 1:**
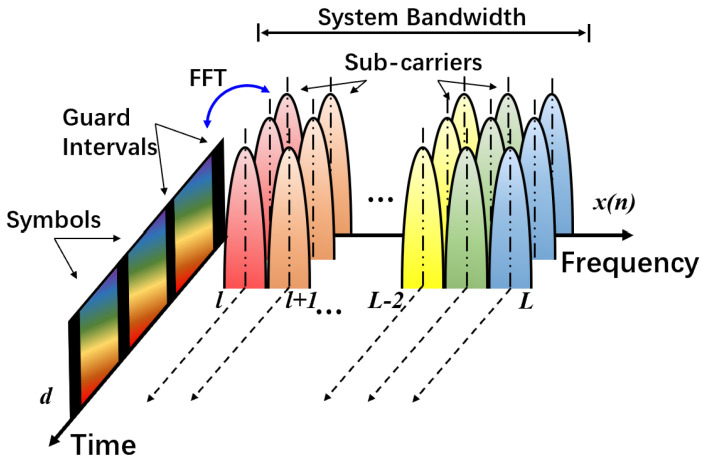
Time & frequency transformation of multi-carrier waveform.

Denote *s* as the symbol index, *l* as the sub-carrier index, *L* as the number of sub-carrier and *n* as the time index. Define $d_{s,l}\in C$ as the source symbol. Then the general waveform framework based on the concept of OFDM can be expressed as

(1) $$x\left(n\right)=\sum_{s=-\infty }^{\infty }\sum_{l=0}^{L-1}d_{s,l}\cdot p\left(n-sL\right)\cdot e^{j2\pi lF(n-sL)},$$

where p(n) is the prototype filter and $e^{j2\pi lF(n-sL)}$ is the Fourier transformation kernel corresponding to the value of frequency shift in the *l*-th sub-carrier, where *F* is the reciprocal of *L*, i.e., F=1/L. The above-mentioned time and frequency transformation relationship is also illustrated in Figure 1.

### 2.2. Performance Metrics

Some major performance metrics of waveforms, i.e., PAPR and throughput, are discussed in the following.

#### 2.2.1. Peak-to-Average Power Ratio

Denote x(n) as the transmit signal. The performance metric PAPR is defined as the ratio of the peak value to the average value of x(n), which is given by

(2) $$\mathrm{PAPR}=\frac{{\max|x\left(n\right)|}^{2}}{{\mathrm{mean}|x\left(n\right)|}^{2}}.$$

Due to the limited dynamic range of the power amplifier in high frequency transmission, the signal with high PAPR suffers from non-linear distortion. Therefore, PAPR is a key indicator of the power efficiency of the multi-carrier system.

The OFDM signal is composed of multiple independently modulated sub-carrier signals. When the phases of the sub-carriers are the same or similar, the superimposed signal will be modulated by the same initial phase signal, resulting in a larger instantaneous power peak value, which further causes a high PAPR. Due to the high PAPR, the OFDM signal can easily reach the boundary of the dynamic range of a power amplifier [26]. This can cause unexpected distortion, especially in high frequency transmissions, and may even cause inter-carrier interference due to the leakage. This has hindered the application of OFDM-like waveforms in mmWave and THz communication systems [27,28].

#### 2.2.2. Throughput

Throughput refers to the amount of data that are successfully transmitted per unit of time. The throughput of a transmission system is defined as

(3) Throughput=(1−BLER)×TBS/T,

where BLER denotes the block error rate, TBS denotes the transport block size and *T* is the period of transmission data block.

The throughput of OFDM systems depends on the modulation order, channel coding rate and the number of occupied sub-carriers. However, for future-oriented new scenarios, the transmission rate supported by OFDM is still not high enough. In order to provide good performance for complicated application scenarios, waveforms with low PAPR and high spectrum efficiency are required for high-frequency wireless transmission.

## 3. Proposed IOTA-Based FTN-DFT-s-OFDM Waveform Design

We consider improving the spectral efficiency and reducing the PAPR on the basis of OFDM. Based on the commonly deployed DFT spreading technique, we further enhance the spectral efficiency with FTN signaling, where FTN acceleration can reduce the transmission time of each symbol. However, due to the introduction of FTN, ISI is unavoidable. Therefore, the design of prototype filter with better focusing capability is studied.

In Section 3.1, we present the design of the waveform framework. The corresponding receiving method is illustrated in Section 3.2.

### 3.1. Transmission Waveform Design

In this part, the components of the proposed transmission waveform are introduced, including DFT spreading, FTN signaling and IOTA filter design, respectively.

#### 3.1.1. DFT Spreading

DFT spreading can be regarded as a kind of precoding before OFDM modulation. By deploying DFT spreading on the source symbols, the resulted waveform, termed DFT-s-OFDM, can hold the characteristics of single carrier waveform. For the *s*-th OFDM symbol, we denote $c_{s}={\left[c_{s,0},c_{s,1},\ldots ,c_{s,m},\ldots ,c_{s,M-1}\right]}^{T}$ as the *M*-dimensional source symbol vector. Then the transformed signal for the *s*-th symbol duration after *M*-point DFT is given by

(4) $$d_{s,l}=\frac{1}{\sqrt{M}}\sum_{m=0}^{M-1}c_{s,m}\cdot e^{-j2\pi ml/M},\;\left(l=0,1,\ldots ,M-1\right).$$

Substituting (4) into (1), we have the overall transmit signal for DFT-s-OFDM. The inherent single carrier structure make the above signal a promising enabler of high-frequency communication.

#### 3.1.2. FTN Signaling

Given the discrete transmit symbol (1), FTN signaling aims to transmit each symbol with a shorter time interval to reserve the time resources. We reuse the above notations, and denote x(n) as the input to the FTN module. Denote $T_{0}$ as the normal symbol transmission interval (i.e., the interval under Nyquist sampling), the exact symbol interval after FTN signaling is given by $T=\alpha T_{0}$, where α is the acceleration factor ranging within [0,1]. The acceleration factor α represents the compression degree of FTN, where the FTN waveform under small α has large spectral efficiency. Denote g(t−nT) as the shaping filter and overall received signal is derived by

(5) $$y\left(t\right)=\sqrt{E_{s}}\sum_{n}x\left(n\right)g\left(t-nT\right)+n\left(t\right),$$

where n(t) denotes the additive white Gaussian noise (AWGN), g(t−nT) denotes the shaping filter and $E_{s}$ denotes the average power of the transmitting signal.

FTN can be defined as pulses that are shorter in time and thus no longer orthogonal. We can easily observe that smaller α results in a higher data rate. However, due to the non-orthogonality of pulses in FTN signaling, there are overlaps among carriers, and ISI appears. The performance of communication systems thus degrade because of the presence of ISI. In the next, we introduce IOTA filter, which helps to resolve the ISI brought by FTN signaling.

#### 3.1.3. IOTA Filter Design

IOTA is obtained from orthogonal Gaussian pulse which holds the optimal time-frequency focusing property. The IOTA pulses are generally called root-Nyquist self-transform pulses, where self-transform means that the beginning of the derivation is Gaussian-shaped pulse and root-Nyquist means the end of the derivation is orthogonal pulse.

IOTA filter orthogonalizes the Gaussian filter, and has the same good time-frequency focusing performance as the Gaussian filter [29]. It can be used to reduce the out-of-band emission, PAPR and the ISI brought by FTN signaling.

In the time-domain, the IOTA pulse, denoted as $\xi_{\tau_{0}}\left(t\right)$, can be given by [30]

(6) $$\begin{array}{cc} \xi_{\tau_{0}}\left(t\right)=\frac{1}{2} & \sum_{k=0}^{K-1}\left\{{\bar{d}}_{k,v_{0}}\left[h_{EGF}\left(t+\frac{k}{v_{0}}\right)+h_{EGF}\left(t-\frac{k}{v_{0}}\right)\right]\right\}\cdot \sum_{l=0}^{K}\left[{\bar{d}}_{l,\tau_{0}}\cos\left(2\pi l\frac{t}{\tau_{0}}\right)\right],\\ & -4\tau_{0}\leq t\leq 4\tau_{0}, \end{array}$$

where *K* is a constant parameter for IOTA, $h_{\mathrm{EGF}}\left(t\right)$ represents the extended Gaussian filter (EGF) in the time domain and ${\bar{d}}_{l,v_{0}}$ represents the IOTA coefficients given by

(7) $${\bar{d}}_{l,v_{0}}=\sum_{q=0}^{Q-1}b_{k,q}\cdot e^{-\pi (2q+k)},0\leq k\leq K-1,0\leq q\leq Q-1,$$

with *Q* also being a constant value and $b_{k,q}$ the pre-defined weight coefficients.

The important parameters to generate IOTA filter are listed in Table 1.

The proposed waveform uses an IOTA filter as the prototype filter by replacing p(n) in (1) with (6). We get p(n) by discrete sampling on IOTA filter, with the sampling period the same as DFT-s-OFDM (i.e., T/L), where p(n) can be found by

(8) $$\begin{array}{cc} p\left(n\right)=\frac{1}{2} & \sum_{l=0}^{L-1}\left\{{\bar{d}}_{l,v_{0}}\left[h_{EGF}\left(\frac{nt}{L}+\frac{l}{v_{0}}\right)+h_{EGF}\left(\frac{nt}{L}-\frac{l}{v_{0}}\right)\right]\right\}\cdot \sum_{l=0}^{L}\left[{\bar{d}}_{l,\tau_{0}}\cos\left(2\pi l\frac{nt}{\tau_{0}L}\right)\right],\\ & -4\tau_{0}\leq t\leq 4\tau_{0}. \end{array}$$

**Figure 2:**
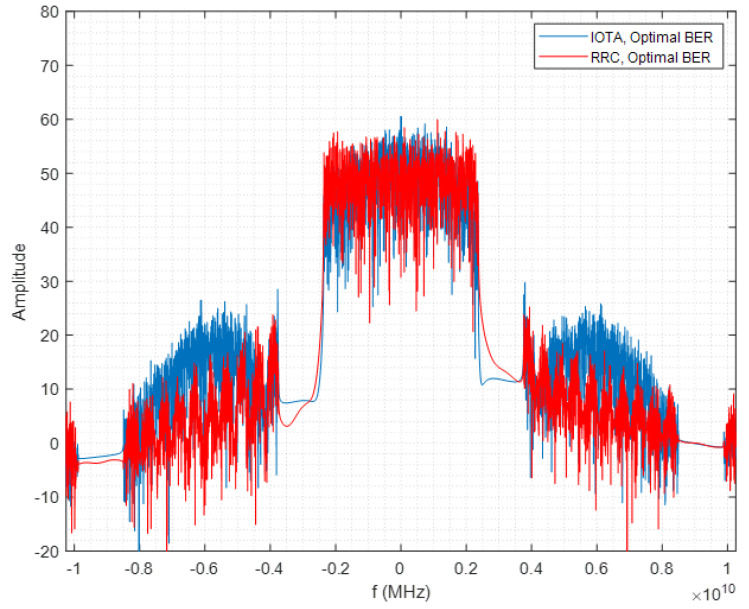
OOB performance of IOTA filter.

IOTA has good performance of focusing on the time and frequency domain, and thus is better at resisting ISI and inter-channel interference (ICI) than a rectangular waveform or root-raised cosine (RRC) filter. IOTA filter also holds similar out of band (OOB) performance as the RRC filter as shown in Figure 2. This indicates that no additional bandwidth is required for deploying IOTA.

### 3.2. Receiver Design

Even with an IOTA filter, the existence of ISI still requires a more complicated receiver to recover the original transmission signals. In the sequel, we design the receiving algorithm for the proposed waveform based on frequency-domain equalization (FDE). The receiver design should lay the basis for the conventional DFT-S-OFDM waveform while considering the specific characteristics of FTN and IOTA filters.

Shiya Sugiura [31] proposes a frequency domain equalization (FDE) receiver structure based on minimum mean square error (MMSE), which can achieve low demodulation complexity, especially for long channel FTN schemes. Specifically, in the FDE scheme, a short cyclic prefix (CP) is added to each transport block and a finite-tap cyclic matrix is adopted to approximate the ISI generated by FTN signaling. Thus, an efficient FFT method and a low-complexity MMSE detection algorithm can be used in the receiver. FDE can achieve near-optimal BER performance without increasing demodulation complexity and power consumption.

In the AWGN channel, the output signal after a matched filter given (5) can be expressed as

(9) $$\hat{y}\left(t\right)=y\left(t\right)*g\left(t\right)=\sqrt{E_{s}}\sum_{n}x\left(n\right)\hat{g}\left(t-n\tau T\right)+\eta \left(t\right),$$

where $\hat{g}\left(t\right)=\int g\left(\tau \right)g^{*}\left(\tau -t\right)d\tau$, $\eta \left(t\right)=\int n\left(\tau \right)g^{*}\left(\tau -t\right)d\tau$ and $E_{s}$ represents the average power of transmitting signal. Assuming that the time synchronization is perfect between the transmitter and receiver, the sampled value of the *k*-th signal at the receiver can be written as

(10) $$\begin{array}{cc} {\hat{y}}_{k} & =\hat{y}\left(k\tau T\right)\\ \; & =\sqrt{E_{s}}\sum_{n}x\left(n\right)\hat{g}\left(k\tau T-n\tau T\right)+\eta \left(k\tau T\right)\\ \; & =\sqrt{E_{s}}x\left(n\right)\hat{g}\left(0\right)+\sqrt{E_{s}}\sum_{n\neq k}x\left(n\right)\hat{g}\left(k\tau T-n\tau T\right)+\eta \left(k\tau T\right), \end{array}$$

where the first part is the symbol at present, the second part is ISI and η(kτT) is the zero-mean random Gaussian variable.

**Figure 3:**
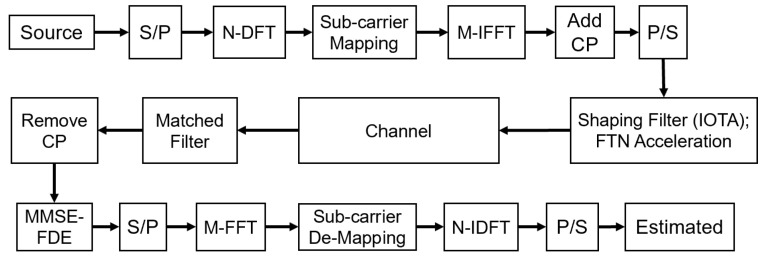
The transceiver design of the proposed waveform.

Figure 3 is the transceiver structure of MMSE-FDE scheme. A CP with length 2v is first added after *N* modulated symbols to realize the symbol transmission based on the block. Then, the first and last *v* receiving signal samples are removed from the entire N+2v samples, and we obtain a received signal block with length *N*, which can be expressed as

(11) $$\begin{array}{cc} \; & \hat{y}={\left[{\hat{y}}_{1},\ldots ,{\hat{y}}_{N}\right]}^{T}\in C^{N}\\ \; & =Gx+\eta , \end{array}$$

where $x={\left[x_{1},x_{2},\ldots ,x_{n}\right]}^{T}$ is the transmitting signal and $\eta ={\left[\eta_{1},\eta_{2},\ldots ,\eta_{N}\right]}^{T}$ is the corresponding channel noise component. The k−th row of tap coefficient matrix $G\in R^{N\times N}$ is

(12) $$G=\left[\begin{array}{ccccccccc} g(-v\tau T) & \cdots & g(v\tau T) & 0 & \cdots & \cdots & \cdots & \cdots & 0\\ 0 & g(-v\tau T) & \cdots & g(v\tau T) & 0 & \cdots & \cdots & \cdots & 0\\ 0 & 0 & g(-v\tau T) & \cdots & g(v\tau T) & 0 & \cdots & \cdots & 0\\ \vdots & \ddots & \ddots & \ddots & \ddots & \ddots & \ddots & \ddots & \vdots \\ 0 & 0 & 0 & \cdots & 0 & g(-v\tau T) & \cdots & g(v\tau T) & 0\\ g((2v-1)\tau T) & \cdots & g(v\tau T) & 0 & \cdots & 0 & g(-v\tau T) & \cdots & g((2v-2)\tau T)\\ g((2v-2)\tau T) & \cdots & g(v\tau T) & 0 & \cdots & 0 & g(-v\tau T) & \cdots & g((2v-3)\tau T)\\ \vdots & \ddots & \ddots & \ddots & \ddots & \ddots & \ddots & \ddots & \vdots \\ g(0) & \cdots & g(v\tau T) & 0 & \cdots & 0 & g(-v\tau T) & \cdots & g(-\tau T) \end{array}\right].$$

Due to the cyclic structure of matrix G, singular value decomposition can be done as $G=Q^{T}\Lambda Q^{*}$, with $Q\in C^{N\times N}$ being the eigenmatrix of G, Λ being a diagonal matrix and elements in the i−th row being the corresponding FFT coefficients. DFT vector can be used to derive the element in the l−th row and k−th column of Q.

To reduce the computational complexity, the considered FDE algorithm aims to equalize the time-domain receiving signal in the transform-domain with the eigenmatrix Q, which is derived as

(13) $$\begin{array}{c} {\hat{y}}_{f}≃Q^{*}\hat{y}=\Lambda Q^{*}x+Q^{*}n=\Lambda x_{f}+n_{f}, \end{array}$$

where $x_{f}$ and $n_{f}$ refer to the transformed signal and noise vector, respectively. Then the equalization matrix $W\in C^{N\times N}$ is derived based on Λ according to the MMSE criteria, with $\omega_{\left(i,i\right)}$ and $\lambda_{\left(i,i\right)}$ the i−th rows of the diagonal matrix W and Λ, respectively. Here, $\omega_{\left(i,i\right)}$ can be expressed as

(14) $$\begin{array}{c} \omega_{\left(i,i\right)}=\lambda_{\left(i,i\right)}^{*}/\left({\left|\lambda_{\left(i,i\right)}\right|}^{2}+N_{0}\right) \end{array}$$

Finally, we get the estimated value $\hat{x}$ of the transmit symbol $x={\left[x_{1},x_{2},\ldots ,x_{n}\right]}^{T}$ as

(15) $$\hat{x}=Q^{T}W{\hat{y}}_{f}=Q^{T}W\left(\Lambda x_{f}+n_{f}\right).$$

The computational complexity of the FDE receiver includes three parts: FFT complexity, weight calculation complexity and MMSE algorithm complexity. (13) uses FFT to transform the received signal into the frequency domain, with its complexity of order NlogN. The calculation of the weight in (14) requires 4N multiplications and the MMSE operation in (15) requires 2N multiplications, whose complexity are both of order *N*. Thus, the proposed receiver has the overall computational complexity of O(NlogN+N). The complexity of the FDE receiver is only related to the receiving block length *N*, but not to the FTN acceleration factor α, filter type, or tap length. Therefore, the receiver complexity of the the IOTA-based scheme proposed in this paper is the same as that of the RRC-based NOW scheme. Compared with the time-domain equalization algorithm, whose complexity exponentially increases with tap length, the FDE receiving algorithm used in this paper has lower complexity, especially under high ISI.

Until now, we finish the signal demodulation and successfully cancel the ISI. What is worth noticing is that FDE has relatively low complexity and can similarly achieve the detecting performance of optimal receiver.

## 4. Simulation Results and Analysis

In this section, we implement the proposed IOTA-based FTN-DFT-s-OFDM and evaluate the PAPR, BER and throughput performance under various MCS settings and acceleration rates. Link-level Monte-Carlo simulations are conducted to valuate the performance gain of the proposed scheme over DFT-s-OFDM and non-orthogonal waveform (NOW) [32], which adopts RRC pulse as the prototype filter of FTN waveform. The main simulation parameters are presented in Table 2.

### 4.1. PAPR

In this section, PAPR performance is compared within different MCSs, each with different values of acceleration factor α.

**Figure 4:**
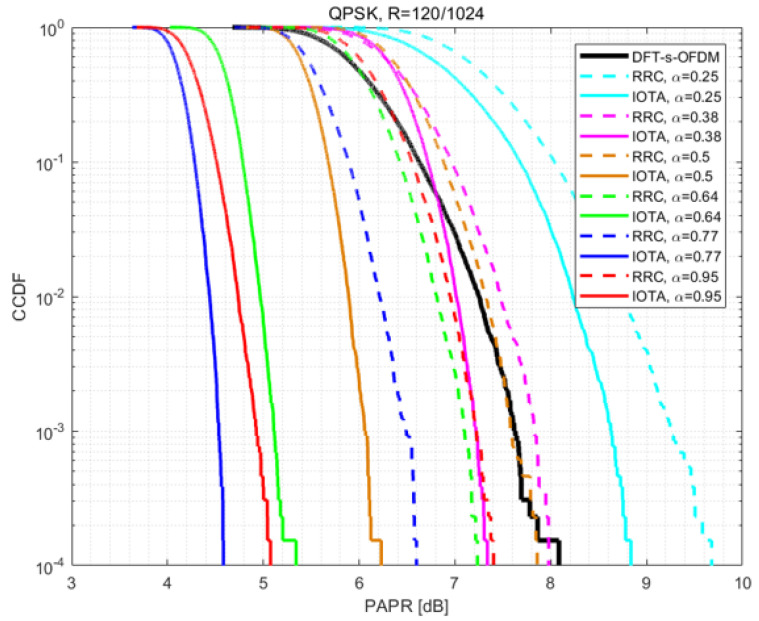
Performance of PAPR based on QPSK with R=120/1024.

**Figure 5:**
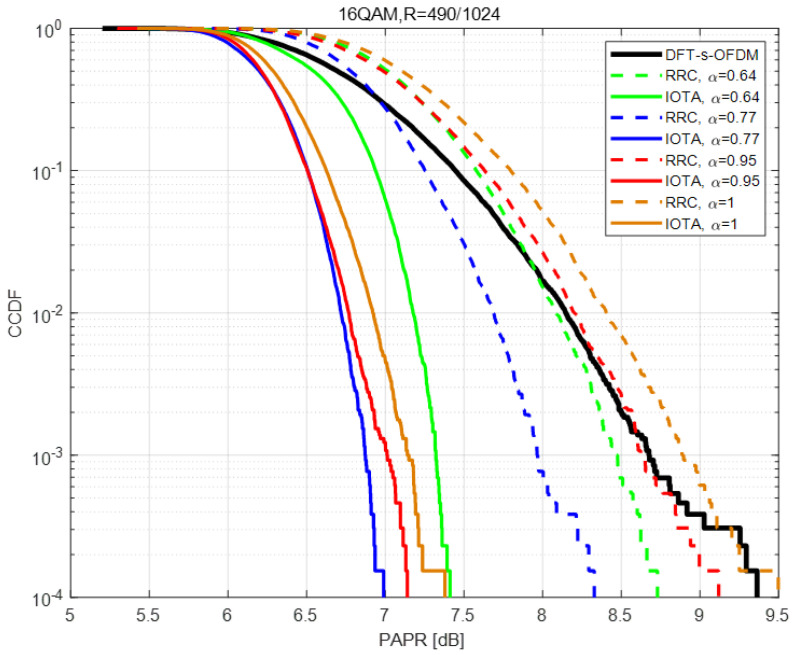
Performance of PAPR based on 16QAM with R=490/1024.

**Figure 6:**
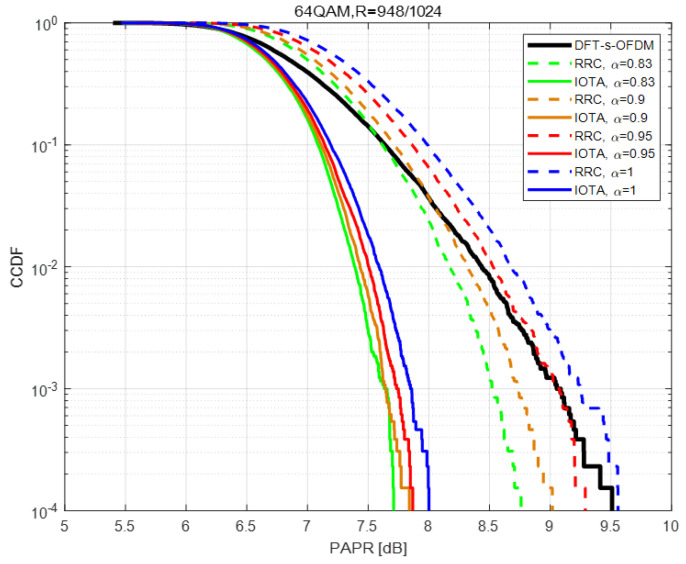
Performance of PAPR based on 64QAM with R=948/1024.

Figure 4, Figure 5 and Figure 6 show the PAPR performance based on QPSK under various MCSs. From the figures, we can conclude that no matter what filter is used (i.e., IOTA or RRC), the PAPR of FTN first decreases and then increases with the decrease of the acceleration factor α, and the PAPR of FTN reaches the optimal value when the acceleration factor is about 0.75–0.85. For higher modulation orders, higher spectral efficiency can be expected, and thus higher PAPR values can be seen. However, PAPR performance has little relation with the coding rate *R* when modulation orders are the same.

Besides, in the simulation process we have, in total, nine MCSs, each with nine indexes (i.e., we have 81 cases actually). The corresponding modulation order, coding rate and spectral efficiency are listed in Table 3, including the corresponding value of α with experimentally best PAPR performance and PAPR gains based on IOTA in each case. Denoted modulation order as Mod, coding rate as *R*, spectral efficiency as SE, α for best PAPR on IOTA as best α -IOTA and α for best PAPR on RRC as best α -RRC.

**Figure 7:**
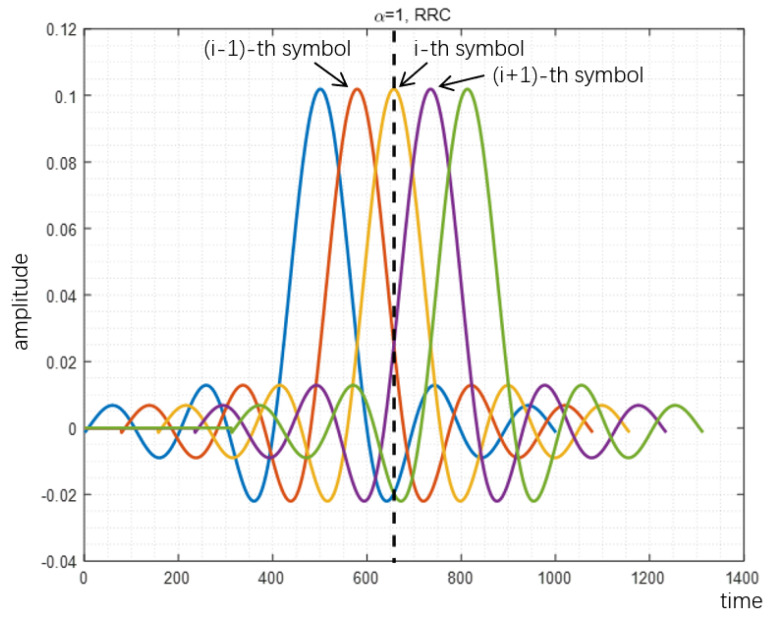
Peak value of RRC filter-based signal with α=1.

**Figure 8:**
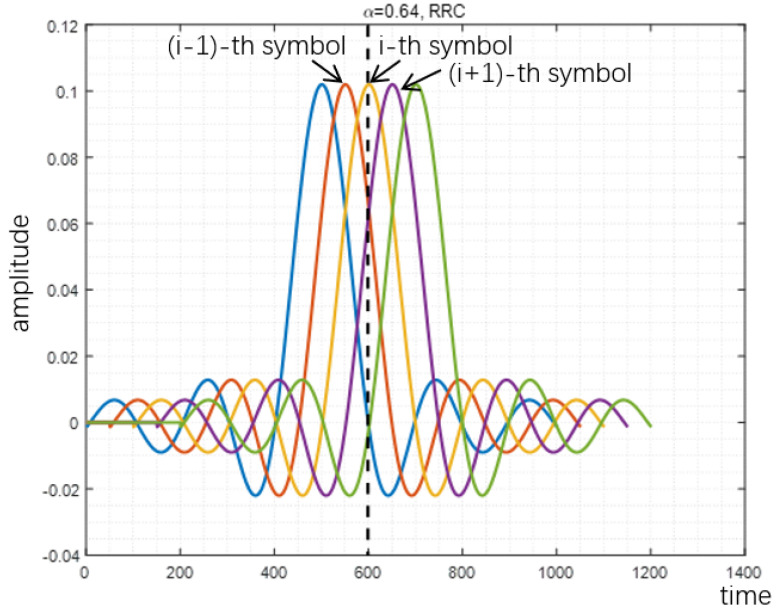
Peak value of RRC filter-based signal with α=0.64.

**Figure 9:**
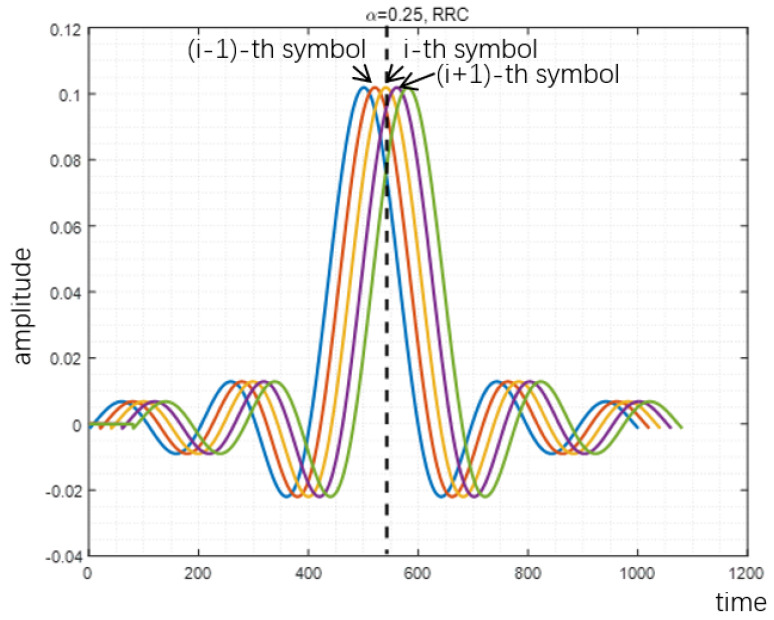
Peak value of RRC filter-based signal with α=0.25.

The reason why proposed method can reduce PAPR is analyzed in Figure 7, Figure 8 and Figure 9. When the acceleration factor α of FTN is large, the degree of compression is relatively low. With the increase of compression degree, the average value increases continuously. The increment of peak value mainly depends on the size of the tails of the pulse. When FTN is further compressed, the size of the peak part is mainly determined by the central envelope of other pulses. In this case, the peak value increases sharply with the increase of the compression degree.

**Figure 10:**
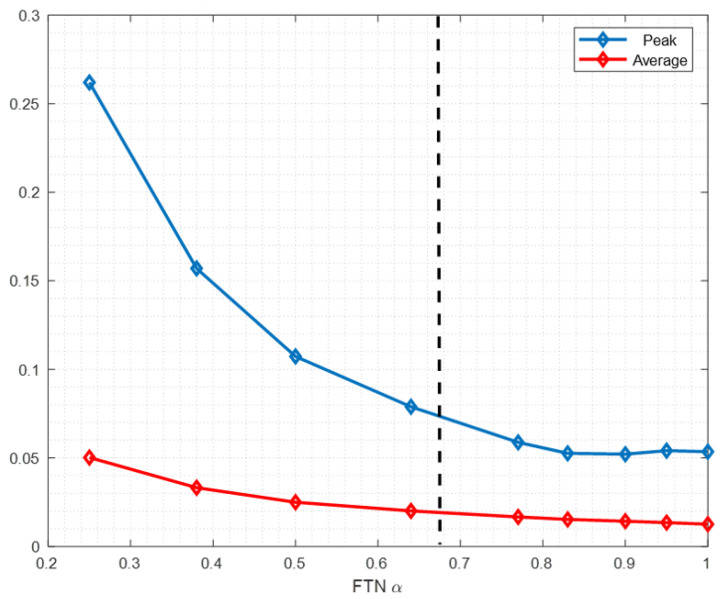
Comparison of peak and average value of FTN under different acceleration factor α.

Comparison is made between the peak value and average value of the FTN waveform, which is shown in Figure 10. As the FTN acceleration factor α decreases, the average value keeps increasing, whereas the peak value first decreases and then slowly increases and finally increases sharply. Therefore, the PAPR of FTN first decreases and then increases accordingly. This explains the observations of PAPR performance in Figure 4, Figure 5 and Figure 6.

### 4.2. BER

This part compares the BER performance of IOTA and non-orthogonal waveform (NOW), based on QPSK and 16QAM with different value of coding rate *R* and acceleration factor α. NOW denotes the algorithm of FTN-DFT-s-OFDM signaling based on the RRC filter.

**Figure 11:**
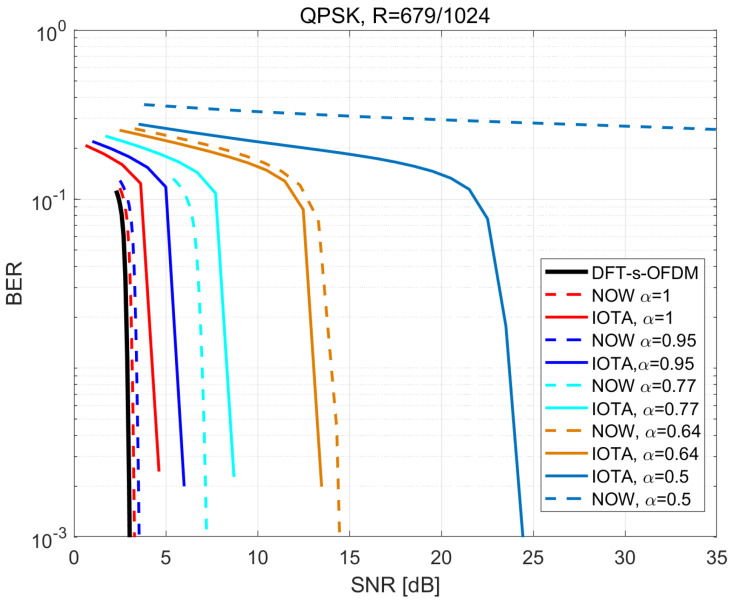
Performance of BER based on QPSK with R=679/1024.

**Figure 12:**
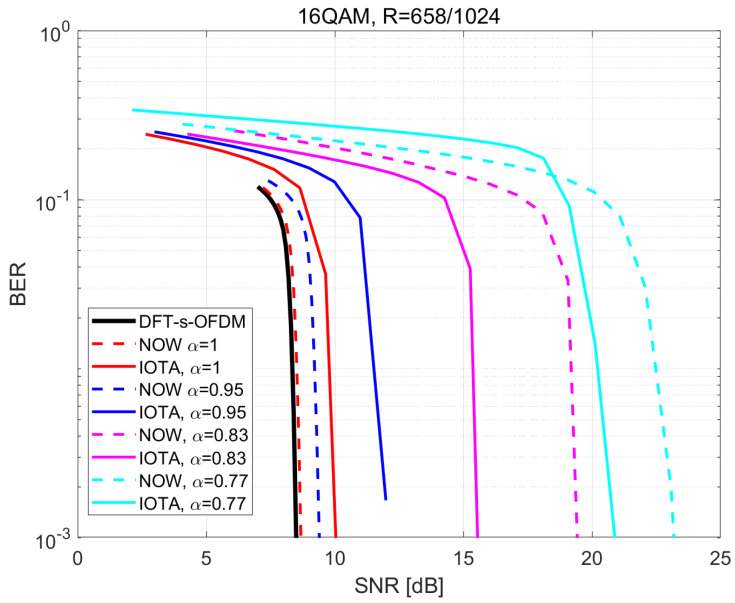
Performance of BER based on 16QAM with R=658/1024.

Figure 11 and Figure 12 illustrate the BER performance based on the modulation order corresponding to MCS2 and MCS5 in Table 3.

From the above two figures, we can derive that the BER performance of IOTA will exceed that of the RRC under small values of α, and also the algorithm based on IOTA can support smaller α. When higher spectral efficiency is achieved with higher modulation orders, BER performance based on IOTA shows a slower decreasing trend compared with RRC. Thus, we can arrive at the conclusion that IOTA seems to be more suitable for FTN signaling, especially for the case with high spectral efficiency.

Similarly to PAPR performance mentioned above, we give Table 4 listing the BER performance based on IOTA. SNR loss compared to RRC and baseline is listed, as well as the minimum supportable α of IOTA and RRC.

### 4.3. Throughput

In this part, thoughput performance of IOTA and RRC is compared under different α. The throughput is calculated as

(16) Troughput=(1−BLER)∗TBS/(T1×α+T2),

where BLER is block error rate, TBS is the transport block size, which represents the number of bits in each block, T1 is the transmission period of data block (i.e., slot time) and T2 is the transmission period of CP.

**Figure 13:**
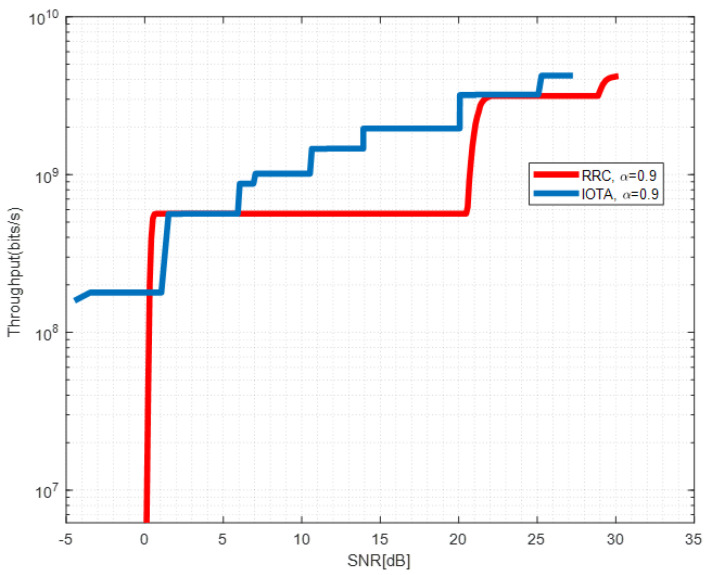
Throughput performance of IOTA and RRC with α=0.9.

**Figure 14:**
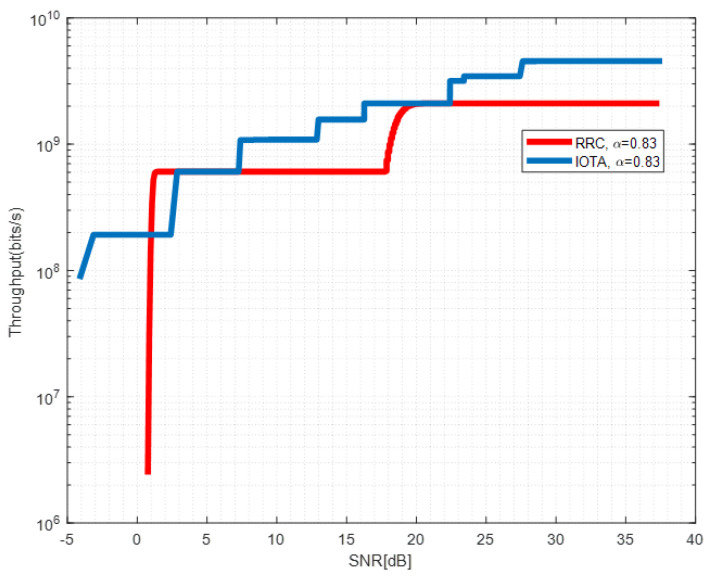
Throughput performance of IOTA and RRC with α=0.83.

**Figure 15:**
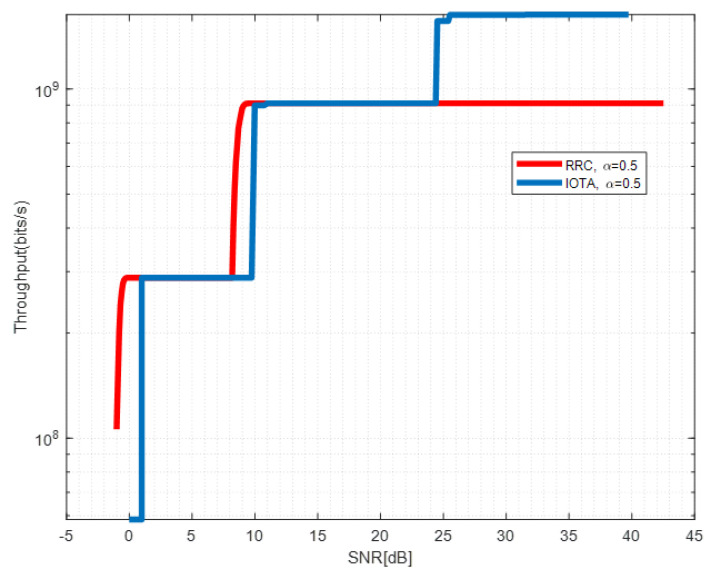
Throughput performance of IOTA and RRC with α=0.5.

Figure 13, Figure 14 and Figure 15 show the throughput performance of systems based on IOTA and RRC filter when different acceleration factors are applied.

By adjusting the MCSs in Table 4, we derive the red line in Figure 13 which depicts the envelope of the achievable throughput of RRC-based waveform under different SNRs. For the RRC-based waveform, due to the ISI brought by FTN, the signal to interference plus noise ratio (SINR) required for successful decoding of the MCS with a larger index can not be fulfilled even with increased SNR up to 20 dB. This makes the achievable throughput maintain a constant value in a large SNR region. However, for the IOTA-based waveform, reduced ISI enhances the SINR and throughput can be enhanced by changing the MCSs. We observe that significant throughput improvement can be seen with SNR between 15–18 dB, which corresponds to the relative SE of MCS 1 and 5 in Table 4. Similar observations can be drawn in Figure 14 and Figure 15. The throughput performance gain of IOTA is mainly due to its effective ISI reduction.

## 5. Conclusions

In order to meet the new requirements of waveforms applied in high frequency communication, where ultra high-speed transmission is one of the vital targets, high spectrum efficiency with reliability is required in the designed waveforms. Based on the OFDM waveform which is basically used in 4G/5G, DFT spreading and FTN signaling are utilized to make the waveform have lower PAPR and higher spectral efficiency, respectively. To further enhance the throughput performance of communication systems, FTN-DFT-s-OFDM waveform based on an IOTA filter is proposed in this paper. The good time-frequency focusing characteristic of the IOTA filter enables the ISI reduction, and thus the transmission performance of the waveform is improved. On the receiver side, we apply an FDE receiver to demodulate the signal, with a complexity of O(NlogN+N). Simulation results have shown that the proposed scheme can offer joint performance gain in terms of PAPR reduction, BER and throughput improvement. Specifically, 3.5 dB gain in PAPR and 50% gain in throughput can be achieved compared with the existing waveforms.

For future work, improvements can be further achieved in the waveform design and receiver design. Spectral efficiency and PAPR performance enhancement can be explored based on other waveforms, such as constant envelope OFDM, filter bank of multi-carrier, orthogonal time frequency space, etc. Moreover, low-complexity iterative design to compact the ISI and ICI is also a promising direction. The state-of-the-art artificial intelligence techniques can also be exploited to enhance the waveform design in an end-to-end fashion [10,34].

## Figures and Tables

**Table 1:** Parameters of IOTA filter [29,30].

Parameters	Specifications
$\tau_{0}$	$1/\sqrt{2}$
$v_{0}$	$1/\sqrt{2}$
*t*	$\left[-2\sqrt{2},2\sqrt{2}\right]$
*K*	15
*Q*	8
$h_{\mathrm{EGF}}\left(t\right)$	$=2^{\frac{1}{4}}e^{-\pi t^{2}}$

**Table 2:** Parameters for Simulation.

Carrier Frequency	70 GHz [33]
Subcarrier spacing (SCS)	Δf=960 kHz
Symbol Interval	T=1.04μs
System bandwidth	800 MHz
DFT size	7792
Guard band	40 MHz
Allocated subcarriers	M=792 (DFT size)
FFT size	1024
Roll-off factor of RRC	β=1/9
	20(α=1)/28(α=0.95)/30(α=0.9)/
RRC taps	32(α=0.83)/34(α=0.77)/42(α=0.64)/
	52(α=0.5)/68(α=0.38)/102(α=0.25)
Up-sampling factor for signal	$K^{\prime }=\alpha K_{0}\left(K_{0}=78\right)$
Channel	AWGN
Channel coding	LDPC
Modulation	QPSK, 16QAM, 64QAM
Baseline Waveforms	DFT-s-OFDM/NOW [32]

**Table 3:** PAPR gains under different MCS cases.

MCS Index	Mod Order	*R* [/1024]	SE	Best α -IOTA	Best α -RRC	PAPR Gain over RRC /dB	PAPR Gain over DFT /dB
0	2	120	0.2344	0.77	0.77	2	3.5
1	2	379	0.7402	0.77	0.77	2	3.5
2	2	679	1.3262	0.77	0.77	1.8	3.5
3	4	340	1.3281	0.83/0.9	0.77	1.6	2.6
4	4	490	1.9141	0.83	0.77	1.3	2.4
5	4	658	2.5703	0.83	0.83/0.77	1.5	2.5
6	6	438	2.5664	0.83	0.83/0.77	0.9	1.9
7	6	719	4.2129	0.83	0.83	1.3	1.7
8	6	948	5.5547	0.83	0.83	1.1	1.8

**Table 4:** Throughput Gains under different MCS Cases.

MCS Index	Mod Order	*R* [/1024]	SE	Min α -IOTA	Min α -RRC	Throughput Gain
0	2	120	0.2344	0.25	0.38	0.52
1	2	379	0.7402	0.38	0.5	0.32
2	2	679	1.3262	0.5	0.64	0.28
3	4	340	1.3281	0.5	0.64	0.28
4	4	490	1.9141	0.64	0.77	0.20
5	4	658	2.5703	0.64	0.83	0.30
6	6	438	2.5664	0.64	0.83	0.30
7	6	719	4.2129	0.77	0.9	0.17
8	6	948	5.5547	0.77	0.9	0.17

## Data Availability

Not applicable.

## Abbreviations

The following abbreviations are used in this manuscript:

OFDM	Orthogonal frequency division multiplexing
DFT	Discrete Fourier transformation
DFT-s	Discrete Fourier transformation spreading
ISI	Inter-symbol interference
ICI	Inter-channel interference
FTN	Faster-than-Nyquist
IOTA	Isotropic orthogonal transform algorithm
PAPR	Peak-to-average power ratio
BER	Bit error rate
CP	Cyclic prefix
MCS	Modulation coding scheme
AWGN	Additive white Gaussian noise
EGF	Extended Gaussian filter
FDE	Frequency domain equalization
MMSE	Minimum mean square error
